# Navigating equity in global access to genome therapy expanding access to potentially transformative therapies and benefiting those in need requires global policy changes

**DOI:** 10.3389/fgene.2024.1381172

**Published:** 2024-04-04

**Authors:** Tsung-Ling Lee, Tsutomu Sawai

**Affiliations:** ^1^ Graduate Institute of Health and Biotechnology Law, Taipei Medical University, Taipei, Taiwan; ^2^ Graduate School of Humanities and Social Sciences, Hiroshima University, Hiroshima, Japan; ^3^ Institute for the Advanced Study of Human Biology (ASHBi), Kyoto University, Kyoto, Japan; ^4^ Centre for Biomedical Ethics, Yong Loo Lin School of Medicine, National University of Singapore, Singapore, Singapore

**Keywords:** genome therapy, health equity, international human rights law, right to health, right to science, global health

## Abstract

In December 2023, the US Food and Drug Administration and the UK Medicines and Healthcare Products Regulatory Agency granted the first regulatory approval for genome therapy for sickle cell disease. This approval brings hope to those suffering from this debilitating genetic disease. However, several barriers may hinder global patient access, including high treatment costs, obtaining informed consent for minors, inadequate public health infrastructure, and insufficient regulatory oversight. These barriers reflect the structural inequalities inherent in global health governance, where patient access often depends on social and institutional arrangements. This article addresses concerns around informed consent, treatment costs, and patient access, and proposes corresponding policy reforms. We argue that these discussions should be framed within a broader global context that considers social and institutional structures, global research priorities, and a commitment to health equity.

## Introduction

Somatic genome editing with the CRISPR-Cas-9 technique is a rapidly evolving research field with considerable potential to ameliorate various debilitating genetic diseases, including sickle cell disease (SCD). This prevalent monogenic disorder chiefly affects individuals of African descent, with the highest disease burden in sub-Saharan Africa. Approximately 1,000 African children are born with this common genetic disease daily, and more than half will die before age five, primarily due to complications from infection or severe anemia. Despite its lower prevalence, SCD also affects Hispanics, South Asians, Caucasians (specifically those from southern Europe), and individuals of Middle East descent, who may carry the trait and live with this debilitating disease. However, the advent of exagamglogene autotemcel—a CRISPR-based gene-editing therapy colloquially known as exa-cel—could soon herald a transformative treatment for SCD, as underscored at the Third International Summit on Human Genome Editing. (Royal Society, 2023).

Although this innovative therapy has received both regulatory approval from the United Kingdom’s Medicines and Healthcare Products Regulatory Agency and the United States’ Food and Drug Adminstration, (GOV.UK, 2023; Kolata, 2023b), its substantial cost may present significant barriers to access, both within these two countries and potentially globally. Issues of accessibility and affordability are pivotal in ensuring the equitable application of this novel therapy to enhance global health. Potential impediments such as countries’ lack of regulatory capacity to evaluate these advanced therapies, a shortage of manufacturing capabilities for genome therapy production, inadequate data infrastructures for the protection of sensitive genome information, health literacy deficits impeding comprehension of treatment benefits and risks, and deficient public health systems and workforce, could globally restrict patient access. These challenges loom over the groundbreaking potential of genome editing therapy.

These regulatory and ethical issues are not unique to sickle cells, but looms over potentially transformative gene therapies for clinic care. These include etranacogene dezaparvovec (Hemgenix) for the treatment of adult hemophilia B, voretigene neparvovec (Luxturna) for retinal dystrophy treatment, onasemnogene abeparvovec (Zolgensma) for pediatric spinal muscular atrophy, and tisagenlecleucel (Kymriah) for treating acute lymphoblastic leukemia (ALL), follicular lymphoma, and B-cell lymphoma, for instance (Henderson et al., 2024).

This article brings these concerns into focus and proposes corresponding policy reforms by drawing attention to the regulatory and ethical challenges confronted in SCD genome therapy as an example to highlight these global health concerns. By assessing potential barriers during the stage of research and development, regulation, manufacturing/commercialization, and delivery/clinical care, we establish an ethical and legal framework guiding policy reforms at national and international levels whilst also outlining pertinent initiatives currently in progress. We developed the framework with generalized principles such that it can be applied to other genome therapies as they develop. By aligning these ethical and governance principles with international human rights law, we identify the appropriate legal foundations for policy actions and reforms.

Given the high prevalence of SCD in low-resource countries, global accessibility of this novel therapy must transcend mere availability. Through an equity lens, we draw on international human rights law–specifically the right to health and the right to science–to demarcate the responsibilities of national governments and international agencies in promoting equitable access to genome therapy.

We propose that increased public investments in health and health-related services could amplify individual wellbeing and autonomy, fostering economic development in the process. This implies that concerted planning and investment in public health infrastructures should complement international research collaborations, thereby enhancing local clinical research capacity and promoting equitable outcomes (Fogarty International Center, 2023).

## Barriers to equitable access

### Research and development phase

**FIGURE 1:**
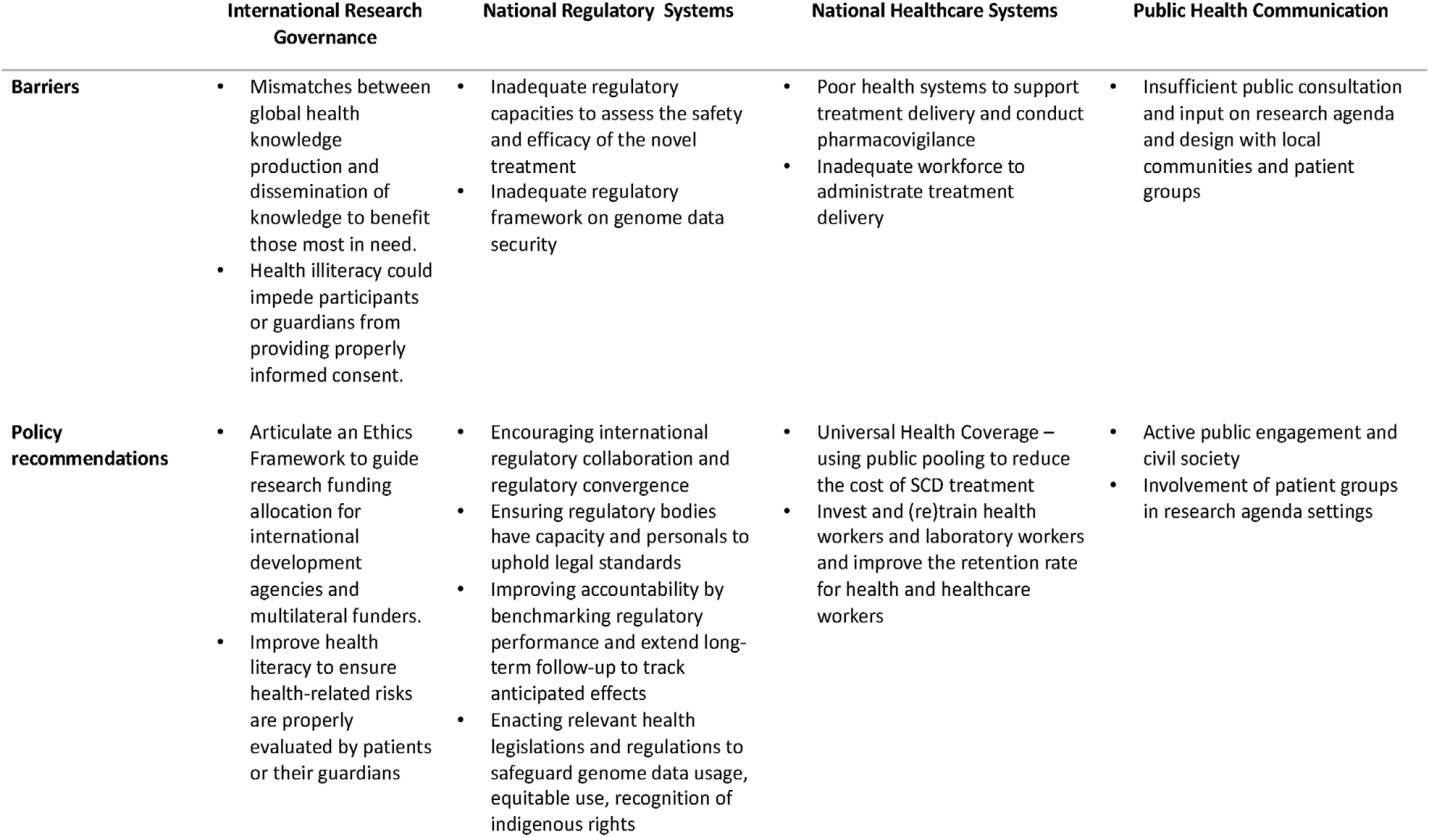
Barriers and policy recommendations.

During the research and clinical phases, a foremost concern is the complexity of obtaining informed consent, especially when treating minors with this therapy. In the pediatric context, the informed consent process must consider the child’s understanding and ability to make decisions about their health. (COMMITTEE ON BIOETHICS, 2016). Additional efforts must be made to ensure that parents or guardians, as proxies, are fully equipped with comprehensive and understandable information about the potential risks and benefits of the therapy, long-term implications, and alternative treatment options (Figure 1). (Spriggs, 2023)

Relatedly, the prospective availability of SCD genome therapy in the United States underscores a prevalent incongruity between knowledge production and dissemination. Indeed, the potential availability of SCD genome therapy in the United States underscores a prevalent disparity between knowledge production and dissemination. The high cost of genome therapy limits the treatment to individuals with substantial financial means or support, despite the transformative impacts this knowledge could have on individual and public health. This gap indicates that this revolutionary therapy may not reach the populations who stand to benefit most significantly. Consequently, addressing accessibility and affordability hurdles should be integral to novel biotechnology’s research and development phase (Nature, 2023).

Equally, at the global level, there has been a significant delay between the introduction of new technologies in affluent countries and their eventual dissemination to less wealthy nations when costs of these technologies decrease historically (WHO, 2022a). This lag period, often extensive, has created a glaring global disparity in access to technology and its benefits. A global survey suggests that genome therapies for rare diseases will likely become standard care around 2036 (Braga et al., 2022). However, if the gap between the production and dissemination of genomic technology is not addressed globally, this could potentially continue to prevent resource-poor countries from benefiting from these genome therapies more broadly.

Consequently, the newly established Science Division under the World Health Organization (WHO) has begun to address equitable access issue and recognizes that the global health agency has a role in promoting affordable access to genome therapies globally (WHO, 2022b).

### Regulatory phase

Cell and gene therapies harbor unique development processes, where requirements for manufacturing, quality control, nonclinical assessment, clinical development, and post-marketing surveillance may diverge significantly from regulatory prerequisites for other pharmaceutical or biotherapeutic products. Across the world, countries possess disparate capacities to evaluate and assess cell and gene therapies. The United States, European Union, and Japan have instituted specific regulatory frameworks for gene therapies (Halioua-Haubold et al., 2017). The European Medicines Agency and the US Food and Drug Administration have issued distinct guidelines or guidance documents for these products (FDA, 2024; European Medicines Agency, 2024).

In contrast, over 90% of National Medicines Regulatory Authorities (NMRAs) in Africa possess little to no capacity to evaluate general medicinal products, with only 7% demonstrating moderately developed regulatory capabilities (Ncube et al., 2021). Complicating these processes are high turnover rates and a dearth of qualified regulatory professionals. While most African countries possess policies supporting medical product regulations, a mere 15% of the NMRAs possess the legally mandated authority to conduct functions in marketing authorization, pharmacovigilance, post-market surveillance, quality control, and clinical trial oversight (Ncube et al., 2021). Given the specialized knowledge and expertise required for cell and gene therapy products, the disparities in legal mandates, regulatory vacuums, and under-resourced regulatory bodies may undermine countries’ ability to conduct regulatory oversights, subsequently decelerating patient access in these countries (Figure 1).

### Manufacturing phase

Genome therapy necessitates the procurement and application of high-quality raw materials, potentially involving human and animal-derived materials (Harrison et al., 2018). The sourcing of these materials outside national borders could pose challenges due to the potential lack of legislation governing importation requirements. This regulatory gap could compromise the safety and affect the quality of genome therapy. For instance, the Constitution of the Republic of South Africa enshrines the right to healthcare, and new drugs are registered through the South African Health Products Regulatory Authority. However, the country lacks specific regulations overseeing the manufacturing or importation of cell and gene therapies to ensure product quality (Kolata, 2023a). Additionally, the specialized facilities and techniques needed for manufacturing and formulating cell and gene therapies necessitate (re)training and educating the local workforce. A lack of manufacturing capacity, expertise, and pertinent legislation governing cell and gene therapy imports may present further barriers to access (Figure 1). (Hendricks et al., 2023)

### Delivery phase

The application of CRISPR-based therapy involves the collection of bone marrow cells from SCD patients. Following genetic editing, these cells are then reintroduced to the patients. This process requires specialized clinical centers, of which there are currently only three serving all of sub-Saharan Africa. Given that many countries grapple with fulfilling basic health needs, administrating genome therapy mandates a healthcare workforce with specialized knowledge and expertise, further taxing the resources of already burdened countries. With 60% of the world’s people living with HIV/AIDS and 90% of annual malaria cases in Africa, (Ncube et al., 2021), ethical questions arise concerning the competition of healthcare needs and the appropriate allocation of limited healthcare resources (Figure 1). The continent countries continue to battle the substantial brain drain of skilled health workers migrating to other countries; for instance, approximately 500 nurses depart Ghana for developed countries monthly (Green, 2023). Therefore, even when genome therapy becomes widely affordable and available, the effective delivery of these treatments hinges on robust health systems and a supportive healthcare workforce.

**FIGURE 2:**
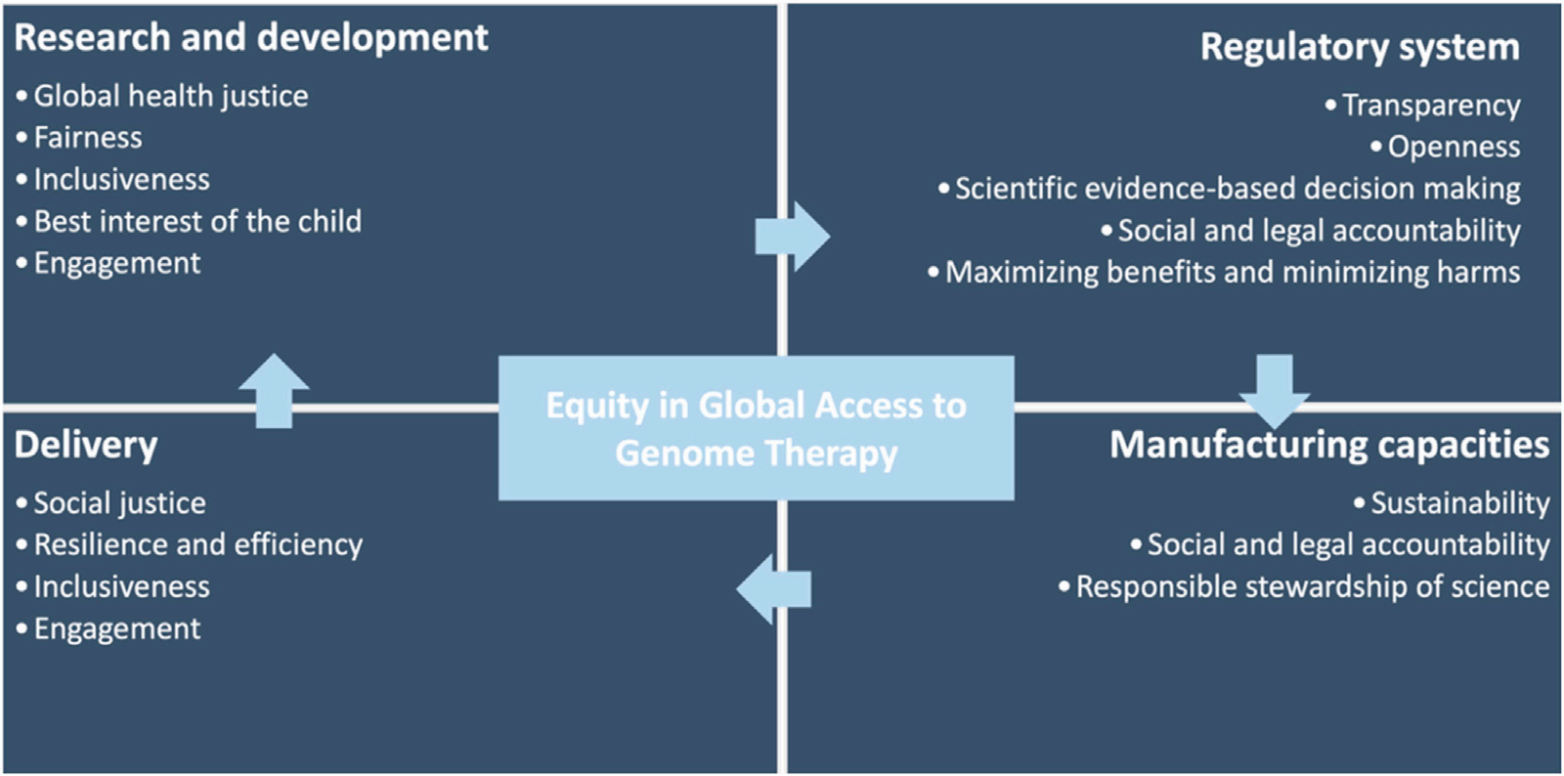
Principles and stages involved in global access to genome editing therapy.

In sum, genome editing therapy’s social and ethical implications extend beyond availability - they raise broader issues around equitable access, benefit-sharing arrangements within the global research paradigm, and the practicalities of delivery (Figure 2). Specifically, the accessibility and delivery of this novel therapy are contingent on the effectiveness of health systems and the robustness of regulatory capacities. These ensure the safety and efficacy of the therapy, which is critical given the disparities in healthcare resources and capacities across nations.

Given the potentially transformative implications of this therapy, including its promise as a cure for SCD, addressing these concerns preemptively is paramount.

## Previous global initiatives

To tackle the public health crisis of sickle-cell anemia, the World Health Assembly and the United Nations General Assembly separately passed resolutions in 2006 and 2008 (WHO, 2006; Resolution A/63/237 of the United Nations General Assembly, 2008). Recognizing the magnitude and severity of SCD as a pressing public health issue, these resolutions called for international collaboration among member states, international organizations, and funding bodies, endorsing basic and applied research. The World Health Organization (WHO) was specifically tasked to foster equitable access to health services, facilitating both prevention and management of the disease. This support dovetailed with assistance for health systems and primary healthcare delivery from United Nations agencies, international institutions, development partners, and various funding programs. Although these resolutions do not have the force of law, they express a collective political stance and carry significant normative authority.

Nevertheless, despite these concerted global efforts, progress toward alleviating the plight of SCD patients has stalled till now. The research on SCD continues to be undervalued compared to other genetic diseases with a similar disease burden (Farooq et al., 2020), creating a chronic funding bias that overlooks the health needs of the global SCD community. SCD patients in high-income countries (HICs), such as the United States, frequently face multiple health inequities due to limited healthcare access and a historical pattern of discrimination, including in the military or workplace environments (Bonham and Smilan, 2019). The high cost of genome therapy will likely serve as an additional hurdle for most individuals living with SCD. The US National Institutes of Health and the Bill and Melinda Gates Foundation are focusing on reducing the costs of these novel therapies in under-resourced countries. The Bill and Melinda Gates Foundation, for example, leads initiatives to lower costs and accelerate the development of sickle cell disease gene therapies by creating a single-shot *in vivo* gene therapy, though this effort presents significant technical challenges and risks (National Academies of Sciences et al., 2023).

Moreover, even if somatic genome editing as a treatment for SCD becomes available worldwide, intellectual property rights–as during the HIV/AIDS pandemic–could be an additional barrier for less affluent countries. Therefore, the discourse surrounding cost and access should be situated within a broader framework encompassing social and institutional structures, global research priorities, and an overarching commitment to health equity.

## Heath equity

The WHO defines health equity as “the absence of unfair, avoidable or remediable differences among groups of people, whether those groups are defined socially, economically, demographically, or geographically or by other dimensions of inequality (WHO, 2024).” This does not suggest that all individuals require equal access to the same resources and types. Rather, it underscores the importance of addressing the unique needs and underlying challenges faced by underserved and vulnerable populations in a way that enhances their wellbeing. Ensuring equitable access to somatic genome editing therapy is crucial in this context. This goal demands careful examination of benefit-sharing arrangements within the domains of international research, regulatory, and health system capacities. The imperative for such scrutiny becomes particularly evident, considering that SCD disproportionately affects sub-Saharan African populations. This region, home to 66% of those living with SCD, is characterized by inadequate public health infrastructure, thus underscoring the need for a balanced approach to resource distribution guided by health equity.

## Legal basis for actions

**FIGURE 3:**
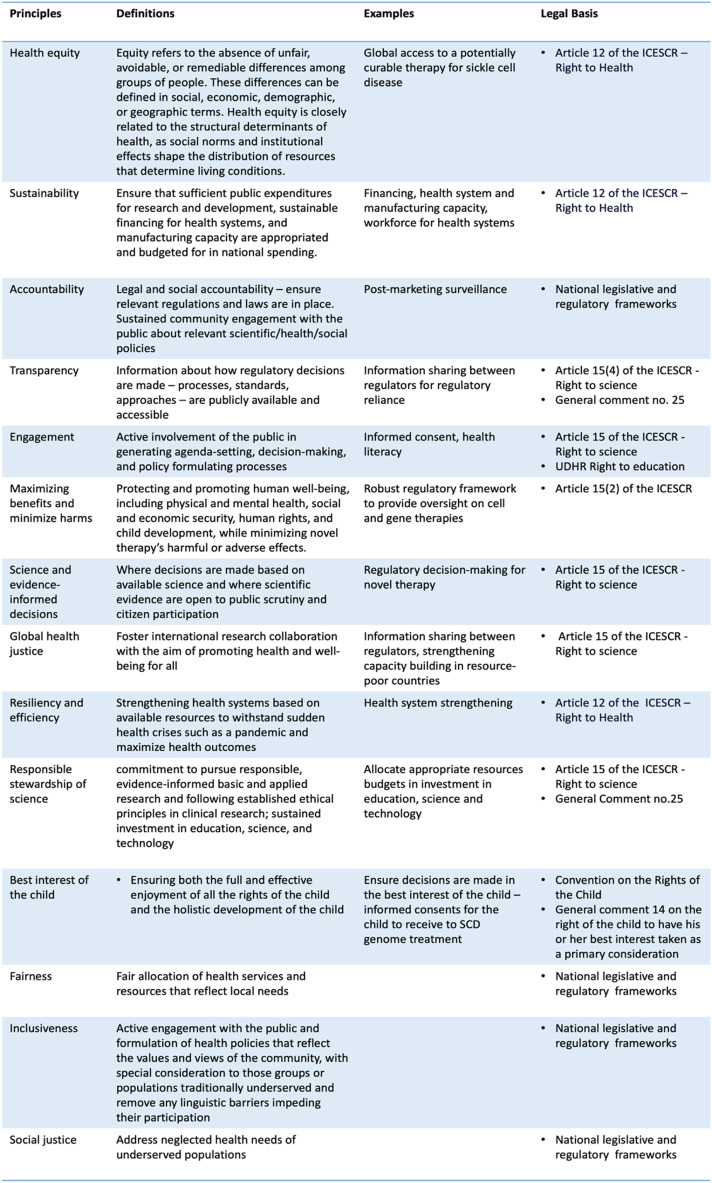
Principles, definitions, examples, and relevant legal bases.

Equity is not merely an ethical principle but also intertwines closely with human rights tenets. Most countries are state parties to the International Covenant on Economic, Social, and Cultural Rights (ICESCR), providing a legal foundation for individuals to demand that their governments progressively ensure their wellbeing. The right to health and science are particularly germane to this discussion (Figure 3).

The right to health, a cardinal human right indispensable for the exercise of other human rights, is enshrined in numerous international and national instruments, including the Universal Declaration of Human Rights, the preamble of the WHO Constitution, the Convention on the Rights of the Child, the African Charter on Human and Peoples’ Rights (1981), and several national Constitutions, such as those in Angola, Botswana, Kenya, and Lesotho.

The right to health enshrined in the ICESCR is subjected to progressive realization and the social-economic circumstances of countries. However, it is essential to emphasize that augmented and sustained public investment in public health infrastructures, and regulatory capacities could foster conditions that empower individuals to become active agents both within and beyond the health sectors, engaging in health and health-related decisions and policies that impact them. Good health facilitates the individual’s pursuit of a good life and becomes an agent for change in developing effective and resilient health systems.

Law plays an integral role in achieving health equity by enabling systemic changes. The ICESCR and other human rights conventions can furnish a foundation for health and health-related legislation to enhance access, availability, and quality of health services. Legal challenges relating to essential medicines, safe maternity care, and equitable treatment of patients with disabilities have triggered policy changes in countries like Uganda (Kruk et al., 2018). Many LMICs have quasi-judicial mechanisms capable of accelerating actions and building political momentum. For instance, the health ombudsman in South Africa was tasked with addressing the systemic failure that resulted in the demise of mental healthcare users (Govender, 2017).

A human rights-based approach to global access to sickle cell disease genome therapy underscores the importance of robust health systems and a regulatory framework to govern cell and gene therapies at the national level. Countries should maximalise available resources and formulate health policies and interventions that progressively enhance the availability, accessibility, and acceptability of healthcare services to meet local needs. Additionally, health interventions and policies should be participatory, non-discriminatory, and transparent, with mechanisms for accountability.

Notably, the *Lancet Global Health Commission* emphasizes that governments should articulate the entitlements of the right to health through a national health plan. This approach will inform the public about their rights and entitlements to health services, establishing accountability through publicly available health system data.

The impending SCD therapy illuminates a challenge government will encounter when allocating resources to balance the demand for expensive, one-time curative treatments with other competing health needs at the population level. This issue is particularly salient in public pooling, where resources are finite and must be distributed to maximize their effects.

To tackle this challenge, some countries, like South Africa, have begun exploring alternative funding mechanisms like the implementation of Universal Health Coverage for gene therapies. Such a mechanism involves pooling resources from government programs to finance expensive treatments for patients who would otherwise be unable to afford them (Cornetta et al., 2022). Through open communication and public involvement in health policies, governments can ensure that resource allocation is fair and equitable.

Similarly, the public has a right to participate in formulating science policy through the right to science. Article 15(1)(b) of the ICESCR recognizes “the right of everyone to enjoy the benefits of scientific progress and its applications.” General Comment 25–adopted by the human rights treaty bodies–provides an authoritative interpretation of the right to science and highlights that state parties have a positive duty to advance science by investing in education, science, and technology and allocating appropriate resources in budgets. Article 15(2) of the ICESCR emphasizes that “the steps to be taken by the States Parties to the present Covenant to achieve the full realization of this right shall include those necessary for the conservation, the development, and the diffusion of science and culture.” It could be posited that the right to science entails sustainable funding for improving regulatory capacity over medicinal regulations and investment in human capital, enabling the public to enjoy the benefits of these novel treatments.

Beyond budgetary measures, fulfilling the right to science requires state parties to adopt legislative and administrative measures to benefit from scientific progress and its application through education policies, grants, and participation in international cooperation with appropriate financing. National efforts to strengthen regulatory and health systems should be communicated to the public such that they understand their health entitlements.

Public health communication forms a core component of the right to science, which entails the government’s duty to enhance scientific literacy and disseminate science in a manner comprehensive to the public. In the clinical setting, this implies ensuring that patients possess adequate health and scientific literacy to understand the potential benefits and risks of SCD genome therapy before providing informed consent. At the societal level, since science is a specialized field of study and an integral part of social, economic, cultural, and political life, the right to science includes translating or curating scientific knowledge into language that the public can easily comprehend (Scheifele et al., 2021). For example, since 2003, Danish law has codified researchers’ duty to disseminate their research and participate in public debates, as the public funds research conducted at universities, either directly or indirectly (Porsdam, 2022).

Lastly, the right to science recognizes international collaboration as a component of fulfilling these rights. To ensure equity and maximize the benefits of scientific knowledge, the process by which knowledge is generated must also be considered. This implies that international research collaboration should occur where its applications will be applied. Engaging local communities and stakeholders can advance and strengthen research, policy, and implementation infrastructure, enabling local communities and patient groups to become co-producers in the knowledge production process, thereby enhancing social accountability.

## Policy reforms: recommendations

### International collaboration

As indicated by available data, international research collaboration tends to be concentrated primarily among HICs. To foster greater collaboration between HICs and low- and middle-income countries (LMICs), it is recommended that funding agencies and international developmental agencies revise their grant assessment criteria. Research initiatives that adopt a global perspective and actively involve local researchers and clinicians should be accorded higher importance during funding assessment. Furthermore, integrating local clinicians into conducting clinical trials in resource-constrained countries could enhance local research capacities. This integration can foster local ownership and facilitate knowledge transfer (WHO, 2021a). To ensure patient access, participants involved in these clinical trials should be given privileged access to genome editing therapy once the regulatory agency approves (WHO, 2021b). Indeed, scholars and practitioners have suggested an overarching ethics framework guiding research funding allocation, which would advance progress in global health (Baumann, 2016a; Ashuntantang et al., 2021; Almeida and Ranisch, 2022). Countries should also invest in and support genome literacy as part of health literacy programs to facilitate meaningful patient engagement in these clinical trials and clinical settings (Figures 1, 3).

### Health systems and regulatory capacities strengthening

The successful delivery of genome editing therapy hinges on the robustness of health systems and regulatory agencies’ capacity to evaluate safety and efficacy and perform pharmacovigilance post-market. In the context of clinical trials conducted in resource-constrained countries, appropriate regulatory frameworks should be in place that reflect ethical norms. Investment in health system strengthening and regulatory capacity enhancement has far-reaching benefits beyond treating SCD. Resilient, responsive, and efficient health systems that optimize available resources can bolster global health crisis management, such as during pandemics. Harmonizing technical requirements for medicines at regional and international levels could facilitate international collaboration among national regulatory authorities and enhance regulatory oversight capacity in these countries, aligning with the Sustainable Development Goals.

The WHO Expert Advisory Committee’s report on developing global standards for governance and oversight of human genome editing contributes significantly to this discourse. Good governance is an iterative process, adapting to both technical and ethical developments in the scientific domain (Baumann, 2016b). As a normative institution, the WHO is pivotal in ensuring regulatory frameworks reflecting ethical norms are established to promote equitable access to novel curative genetic therapies. Furthermore, international research collaborations, critical to global health, could be shaped by international funders altering incentives to boost partnerships between HICs and LMICs. Lastly, the WHO should ensure strategic alignments of the health systems and regulatory capacities by monitoring country-level progress and collaborating with international partners to support these initiatives.

To ensure global equitable access to SCD genome therapies, it is necessary to guide all phases - from research and development to regulatory approval, manufacturing, and delivery - by principles of human rights and health equity. This approach not only broadens access but also ensures that the sustained benefits of SCD genome therapies reach previously underserved populations. Moreover, the framework proposed in this *Perspective* can be applied to emerging genome therapies, thus closing the gap between the HICs and LMICs in accessing these potentially transformative genome therapies.

## Data Availability

The original contributions presented in the study are included in the article/Supplementary Material, further inquiries can be directed to the corresponding authors.
